# Genetics and the Gynecologic Patient

**DOI:** 10.31486/toj.20.0051

**Published:** 2020

**Authors:** Katrina S. Wade, Jacob M. Estes, Richard C. Kline

**Affiliations:** Department of Gynecologic Oncology, Ochsner Clinic Foundation, New Orleans, LA

**Keywords:** *Cowden syndrome*, *genes–BRCA1*, *genes–BRCA2*, *genital neoplasms–female*, *genetic diseases–inborn*, *genetic testing*, *hereditary breast and ovarian cancer syndrome*, *Li-Fraumeni syndrome*, *Lynch syndrome*, *Peutz-Jeghers syndrome*

## Abstract

**Background:** The field of hereditary cancer syndromes and genetic testing for patients and families is a rapidly evolving discipline, with an emphasis on cancer prevention.

**Methods:** We review the literature regarding the most common genetic syndromes associated with gynecologic malignancies and discuss the management of these conditions. We also examine the logistic process surrounding cancer genetic testing and identify some perceived barriers.

**Results:** Five genetic syndromes are known to be associated with gynecologic malignancies: hereditary breast and ovarian cancer, Lynch, Cowden, Peutz-Jeghers, and Li-Fraumeni. Each is associated with varying risks of breast, ovarian, and uterine malignancies. The National Comprehensive Cancer Network guidelines regarding the management of these syndromes are focused primarily on reducing the risk of developing gynecologic malignancies. However, great complexity is involved with genetic testing for patients and their families, and barriers exist for the widespread use and implementation of such testing.

**Conclusion:** Genetic testing is fundamental to primary cancer prevention and to oncologic care. Physicians, payers, and institutions must work collaboratively to maximize genetic testing with the goals of primary cancer prevention and treatment.

## INTRODUCTION

In 2015, President Barack Obama executed an initiative that encouraged the development of precision medicine in all disease sites. Precision medicine is medical care designed to optimize therapeutic benefit for particular groups of patients, especially by using genetic or molecular profiling. One of the models for precision medicine in the field of gynecologic oncology is the genetic predisposition to gynecologic cancers, the most common of which are hereditary breast and ovarian cancer syndrome and Lynch syndrome.^1,2^ Although a high-risk cancer susceptibility mutation is ideally identified before cancer is diagnosed, identifying a mutation in women with cancer helps with tumor biology, prognosis, treatment decisions, clinical trial enrollment, risk assessment and prevention of subsequent malignancies, and cancer risk and prevention for blood relatives. Historically, changes to cancer management based on genetic testing results were lacking. However, beginning in 2014, the US Food and Drug Administration has approved some treatments, including poly (adenosine diphosphate-ribose) polymerase (PARP) inhibitors for the treatment of women with BRCA-mutated ovarian cancer and immunotherapy for endometrial cancers with microsatellite instability consistent with Lynch syndrome.^3-8^

In this article, we review the 5 genetic syndromes associated with gynecologic malignancies and their management. We also highlight some of the logistics surrounding the evolving subject of cancer genetic testing.

## GENETIC SYNDROMES IN GYNECOLOGIC CANCER

### Hereditary Breast and Ovarian Cancer Syndrome

Approximately 15% to 20% of patients with ovarian cancer are BRCA1 or BRCA2 mutation carriers.^9^ These mutations increase a woman's lifetime risk of ovarian cancer up to 40% for BRCA1 carriers and up to 20% for BRCA2 carriers.^9,10^ Estimates of the lifetime risk of breast cancer for BRCA1/2 mutation carriers range from 41% to 90%. In a sample of 488 women with nonmetastatic breast cancer, 6.1% had a BRCA1/2 mutation, but mutation prevalence decreased with age (12% in women diagnosed at 45 years of age or younger and 3% in women diagnosed at 46 years of age or older).^11^ Other genes in the homologous recombination pathway, such as RAD51C, RAD51D, BRIP1, PALB2, and BARD1, may also influence breast and ovarian cancer risk and biology.^12-14^ Patients with an increased likelihood of having inherited a predisposition to breast and ovarian cancer should receive genetic counseling and be offered genetic testing (Table 1).^15^

**Table 1.** Consensus Recommendations for Genetic Counseling and Testing for Hereditary Breast and Ovarian Cancer^15^

Women AFFECTED With the Following Cancers	Women UNAFFECTED With Cancer but With Relatives Meeting the Following Criteria
High-grade epithelial ovarian/tubal/peritoneal cancer Breast cancer diagnosed at ≤45 years Breast cancer and close relative with breast cancer diagnosed at ≤50 years or close relative with epithelial ovarian/tubal/peritoneal cancer at any age Breast cancer diagnosed at ≤50 years and a limited family history Breast cancer and ≥2 close relatives with breast cancer at any age Breast cancer and ≥2 close relatives with pancreatic cancer or aggressive prostate cancer (Gleason score ≥7) Two breast primaries, with the first diagnosed at <50 years Triple-negative breast cancer diagnosed at ≤60 years Breast cancer and Ashkenazi Jewish ancestry Pancreatic cancer and ≥2 close relatives with breast, ovarian/tubal/peritoneal, pancreatic, or aggressive prostate cancer (Gleason score ≥7)	First degree or several close relatives who meet one of the criteria in the left column Close relative carrying a known BRCA1 or BRCA2 mutation Close relative with male breast cancer

Recommendations for the medical management of hereditary breast and ovarian cancer are based on an appreciation of the increased risk for cancer and the early onset of disease. For a woman who is a carrier of a BRCA1/2 mutation, the National Comprehensive Cancer Network (NCCN) guidelines suggest that training in breast awareness with monthly self-examinations should begin at 18 years of age, and semiannual clinical breast examinations should begin at 25 years of age.^16^ Patients between the ages of 25 and 29 years should receive annual breast magnetic resonance imaging (MRI) screenings. The age to begin screening can be individualized if the family history includes a breast cancer diagnosis prior to 30 years of age. Breast MRI screening is preferred to mammogram in the 25- to 29-year age group because of increased breast tissue density. For patients between 30 and 75 years of age, both annual mammogram and breast MRI should be done, alternating every 6 months. For patients older than 75 years, management should be considered on an individual basis.

Counseling for women with a confirmed BRCA1/2 mutation includes discussion of risk-reducing mastectomy and/or risk-reducing salpingo-oophorectomy. Risk-reducing salpingo-oophorectomy is the standard of care for ovarian cancer risk management in BRCA1/2 carriers. For women who do not elect risk-reducing salpingo-oophorectomy, screening with transvaginal ultrasound (TVUS) and the cancer antigen 125 (CA-125) test can be considered every 6 months. However, providers should acknowledge the lack of high-quality data to inform these recommendations, and some patients may reasonably prefer not to undergo screening. In a cohort of 888 women carriers of BRCA1 or BRCA2 mutations who underwent screening with annual TVUS and CA-125, 5 of 10 cancers were diagnosed in women who had had normal screening results 3 to 10 months previously. Eight of the 10 cancers were stage III at diagnosis.^17^

### Lynch Syndrome

Lynch syndrome, also known as hereditary nonpolyposis colorectal cancer, is caused by mutations in DNA mismatch repair (MLH1, MSH2, MSH6, PMS2). Women with Lynch syndrome have increased risk for endometrial and ovarian cancers. The lifetime risks are 20% to 71% and 0% to 13.5% for endometrial and ovarian cancer, respectively.^18^ Women with Lynch syndrome also have a 25% to 50% lifetime risk of colorectal cancer, a somewhat lower risk than their male counterparts.^19^ Other cancer risks include pancreatic, central nervous system, and urothelial malignancies. Patients with an increased likelihood of having Lynch syndrome should receive genetic counseling and be offered genetic testing (Table 2).^15^

**Table 2.** Consensus Recommendations for Genetic Counseling and Testing for Lynch Syndrome^15^

Patients With the Following
Endometrial or colorectal cancer with evidence of microsatellite instability or loss of a DNA mismatch repair protein (MLH1, MSH2, MSH6, PMS2) on immunohistochemistry
First-degree relative affected with endometrial or colorectal cancer who was either diagnosed at <60 years or who is identified to be at risk for Lynch syndrome by a systematic clinical screen that incorporates a focused personal and medical history
First- or second-degree relative with a known mutation in a mismatch repair gene

The NCCN guidelines suggest that carriers of MLH1, MSH2, MSH6, or PMS2 mutations consider total abdominal hysterectomy and/or bilateral salpingo-oophorectomy when childbearing is complete.^16^ No clear evidence supports routine screening for gynecologic cancers in these carriers. Annual endometrial sampling can be considered beginning at 30 to 35 years of age.^20^ Routine TVUS and CA-125 screening is not typically recommended because these screening tests have not been shown to be sufficiently sensitive or specific.^21^ Colonoscopy should be performed every 1 to 2 years starting at age 20 to 25 years or 2 to 5 years prior to the earliest familial cancer diagnosis. Additional cancer screening can be considered based on the specific gene mutation.

### Cowden Syndrome

Cowden syndrome results from a pathogenic mutation in PTEN. The syndrome is associated with multiple hamartomas and/or cancerous lesions in various organs and tissues, including the skin, mucous membranes, breast, thyroid, endometrium, and brain.^22,23^ The lifetime risk for breast cancer for women diagnosed with Cowden syndrome has been estimated at 25% to 50%, with an average age of 38 to 50 years at diagnosis.^22,23^ Thyroid disease, including benign multinodular goiter, adenomatous nodules, and follicular adenomas, has been reported to occur in approximately 30% to 68% of adults with PTEN mutations,^24^ and the lifetime risk for thyroid cancer has been estimated at 3% to 10%.^22^ Macrocephaly and autism spectrum disorders are strongly associated with Cowden syndrome.^25^ Patients with an increased likelihood of having Cowden syndrome should receive genetic counseling and be offered genetic testing (Table 3).^26^

**Table 3.** Consensus Recommendations for Genetic Counseling and Testing for Cowden Syndrome^26^

Major Criteria	Minor Criteria
Breast cancer Endometrial cancer Follicular thyroid cancer Multiple gastrointestinal hamartomas or ganglioneuromas Macrocephaly Mucocutaneous lesions Multiple trichilemmomas Multiple acral/palmoplantar keratosis, pits, papules Mucocutaneous neuromas Multifocal oral mucosal papillomatosis Multiple cutaneous facial papules (verrucous)	Autism spectrum disorder Colon cancer Esophageal glycogenic acanthoses Lipomas Intellectual disability Thyroid adenomas, goiter, nodules Papillary or follicular thyroid caner Renal cell carcinoma Single gastrointestinal hamartoma or ganglioneuroma Vascular anomalies

The NCCN guidelines suggest that women with Cowden syndrome should focus on primary and secondary prevention options for breast cancer because, based on the available literature, breast cancer is the cancer most commonly associated with Cowden syndrome.^16,22,23^ Women with Cowden syndrome should begin regular monthly breast self-examinations at 18 years of age and have semiannual clinical breast examinations beginning at age 25 years. Women with Cowden syndrome should also have an annual mammogram and breast MRI with contrast screening starting at age 30 to 35 years. Risk-reducing mastectomy can be considered. The NCCN guidelines state that endometrial cancer screening does not have a proven benefit in women with Cowden syndrome; however, screening with endometrial biopsy every 1 to 2 years may be considered.^16^ Although no data support risk reduction surgery in women with Cowden syndrome, the option of risk-reducing hysterectomy should be considered. Oophorectomy is not indicated for women with Cowden syndrome alone because Cowden syndrome is not associated with an increased risk of ovarian cancer; however, oophorectomy may be indicated for other reasons.

### Peutz-Jeghers Syndrome

Peutz-Jeghers syndrome is characterized by gastrointestinal polyps; mucocutaneous pigmentation; and elevated risk for gastrointestinal, breast, and nonepithelial ovarian cancers. The syndrome is associated with mutations in the STK11/LKB1 gene. The breast cancer risk in women with Peutz-Jeghers syndrome is 8% at 40 years of age, 13% at 50 years, 31% at 60 years, and 45% at 70 years.^27^ Peutz-Jeghers syndrome is associated with an elevated risk of cervical cancer, most commonly adenoma malignum, with an estimated lifetime risk of 10% in addition to a lifetime risk of 21% for ovarian cancer, most commonly sex cord-stromal tumors.^28^ A clinical diagnosis of Peutz-Jeghers syndrome can be made when an individual meets 2 or more of the following criteria^29^: (1) 2 or more Peutz-Jeghers-type hamartomatous polyps of the small intestine; (2) mucocutaneous hyperpigmentation of the mouth, lips, nose, eyes, genitalia, or fingers; and (3) a family history of Peutz-Jeghers syndrome.

Women with Peutz-Jeghers syndrome are advised to undergo annual screening for cervical cancer (including Pap smear) starting at age 21 years.^30^ As with other hereditary syndromes, screening for ovarian cancer in women with Peutz-Jeghers syndrome is controversial, given the lack of proven benefit in reducing mortality from ovarian cancer.^17^ However, TVUS can be considered beginning at age 18 to 20 years. Monthly breast self-examinations for women with Peutz-Jeghers syndrome starting at age 18 years and annual breast MRI and/or mammography starting at age 25 years are suggested.^30^

### Li-Fraumeni Syndrome

Li-Fraumeni syndrome is a rare hereditary cancer syndrome associated with TP53 mutations. Only approximately 300 families with Li-Fraumeni syndrome are listed in a registry maintained by the National Cancer Institute (NCI).^31^ Li-Fraumeni syndrome is associated with a high lifetime risk for cancer; an analysis from the NCI Li-Fraumeni Syndrome Study (n=286) showed a cumulative lifetime cancer incidence of nearly 100%.^32^ Li-Fraumeni syndrome is characterized by a wide spectrum of neoplasms occurring at a young age, including soft tissue sarcomas, osteosarcomas, premenopausal breast cancer, colon cancer, gastric cancer, adrenocortical carcinoma, and brain tumors.^33^

Classic Li-Fraumeni syndrome diagnostic criteria, based on a study by Li, Fraumeni, and colleagues involving 24 Li-Fraumeni syndrome kindreds, are the following^33,34^: (1) a member of a kindred with a known TP53 mutation; (2) a combination of an individual diagnosed at 45 years of age or younger with a sarcoma and a first-degree relative diagnosed with cancer at 45 years of age or younger, and (3) an additional first- or second-degree relative in the same lineage with cancer diagnosed at younger than 45 years of age or a sarcoma diagnosed at any age.

The NCCN recommendations for breast cancer screening in patients with Li-Fraumeni syndrome are similar to those for BRCA-related breast and ovarian cancer syndrome management, although screening is recommended to begin at an earlier age.^16^ Annual breast MRI and mammography beginning at age 20 years (or at the age of earliest known breast cancer in the family if younger than 20 years) are recommended because of the very early age of onset of breast cancer in families with Li-Fraumeni syndrome.

## GENETIC TESTING

### How Genetic Testing Is Performed

Genetic risk assessment starts with a detailed family history, even for patients who already meet testing criteria, because some women might be best served by testing for multiple genes or syndromes. Genetic testing requires informed consent that should include pretest education and counseling concerning the risks, benefits, and limitations of testing, including the implications of both positive and negative results. False negative results are a risk, and uncertainties are associated with genetic variants of unknown significance. Posttest counseling should include education on risk-reduction strategies.^15^

The family member affected with cancer has the highest likelihood of carrying the mutation and thus should be tested first whenever possible. If more than one family member is affected, members with the following factors should be considered for testing first: youngest age at diagnosis, bilateral disease or multiple primaries, and most closely related to the proband. Testing of unaffected individuals should only be considered when an appropriate family member is not available for testing (eg, the family member is deceased). Another important consideration is that family histories change over time and should be reassessed regularly. For example, patients and family members may have had a hysterectomy and/or ovarian removal which may mask the number of cancers identified in a family.

Genetic assessment for Lynch syndrome, in contrast to BRCA1/2 mutation, may be performed first through tumor testing. Immunohistochemistry for the 4 most common mismatch repair proteins is inexpensive and available through most pathology laboratories. Loss of mismatch repair proteins can direct targeted germline genetic testing. The distinction between germline testing and tumor, or somatic, testing is noteworthy. Germline mutations are present in germ cells at conception and can be inherited by future generations, in contrast to somatic mutations that occur after fertilization and are only perpetuated through mitosis within a specific cell lineage or neoplasm.^35^

The indications for and implications of gene-specific testing vs multiplex/panel testing are another testing issue. Following the discovery of BRCA1/2, it was apparent that these 2 genes were not responsible for all familial cases of breast and ovarian cancer.^12^ Hereditary cancer panel testing is designed to detect mutations in a menu of genes that might contribute to cancer risk and can identify a more robust population of women at increased risk of cancer. The disadvantages of panel testing include a higher rate of variances of unknown significance and findings of deleterious mutations in unexpected genes on a panel test. With the increasing number of cancer susceptibility genes identified, multiplex testing of many cancer susceptibility genes has become increasingly attractive as a cost- and time-efficient testing strategy.

If a hereditary syndrome is identified, the affected patient is faced with communicating the results and recommendations for cascade testing to her relatives. Cascade testing is genetic counseling and testing of blood relatives of individuals who have been identified with specific genetic mutations. Testing protocols and interventions may save lives and improve the health and quality of life for these family members.^36^ However, an obstacle to communicating results is a patient's potential lack of desire to inform relatives, secondary to the emotional burden of cancer or a guilty sentiment about possibly passing a mutation to her children. Therefore, a nonjudgmental environment and practical resources to help patients understand the meaning of a positive test are essential.

### Barriers to Genetic Testing

Most women and their families are empowered by informative results of genetic testing. However, availability of health insurance coverage and other types of health care discrimination for those identified as mutation carriers are concerns. While legal protection against genetic discrimination is not complete,^37^ the following provisions afford some level of protection:

1. The Health Insurance Portability and Accountability Act (1996) specifically states that genetic information in the absence of a current diagnosis of illness does not constitute a preexisting condition.^38^
2. Executive Order 13145—To Prohibit Discrimination in Federal Employment Based on Genetic Information (February 2000) prohibits federal executive branch agencies from discriminating against applicants and employees on the basis of genetic information.^39^
3. The Genetic Information Nondiscrimination Act (May 2008) prohibits group health plans from denying coverage to a healthy individual or charging higher premiums based solely on a genetic predisposition to developing a disease in the future.^40^

Despite strong support from the United States Preventive Services Task Force and the NCCN, genetic testing rates remain low. Genetic testing is universally recommended for women with ovarian cancer, but the testing rates in this population are estimated to be 20% to 30%.^41,42^ Multiple factors may contribute to low testing rates, including lack of physician awareness or time to fully assess family history, lack of patient acceptance, delays and/or denials by third-party payers, variable availability of genetic counseling professionals, lack of reimbursement for genetics professionals, racially and culturally disparate populations, and/or uninsured populations.^43^

Improvements in awareness of hereditary cancer, availability of genetics services, and communication of the importance of genetics evaluation can increase the rates of genetic testing. Strategies for increasing patient access to genetics services include telegenetics (telemedicine technology to provide clinical genetic services); group counseling sessions; integration or embedment of services within specialty oncology clinics; initial genetic testing performed by nongenetic clinicians; quality improvement initiatives; and other clinic support tools such as video education, computer-assisted family history collection, and patient navigation.^44-46^ Reducing financial barriers to genetic testing may improve access to services, especially among medically underserved, uninsured, or impoverished patients.

## CONCLUSION

Many barriers to widespread genetic testing for patients and their families exist, but genetic testing of individuals who meet testing criteria is fundamental to oncologic care. Physicians, payers, and institutions must work collaboratively to maximize genetic testing with the goals of primary cancer prevention and treatment.

## Notes

This article meets the Accreditation Council for Graduate Medical Education and the American Board of Medical Specialties Maintenance of Certification competencies for Patient Care and Medical Knowledge.
